# Reduced sensitivity of SARS-CoV-2 Omicron variant to antibody neutralization elicited by booster vaccination

**DOI:** 10.1038/s41421-022-00375-5

**Published:** 2022-01-17

**Authors:** Xiaoqi Yu, Dong Wei, Wenxin Xu, Yulong Li, Xinxin Li, Xinxin Zhang, Jieming Qu, Zhitao Yang, Erzhen Chen

**Affiliations:** 1 Department of Infectious Diseases, Research Laboratory of Clinical Virology, Ruijin Hospital, Shanghai Jiao Tong University School of Medicine, Shanghai, China; 2 Clinical Research Center, Ruijin Hospital, Shanghai Jiao Tong University School of Medicine, Shanghai, China; 3 Department of Pulmonary and Critical Care Medicine, Ruijin Hospital, Shanghai Jiao Tong University School of Medicine, Shanghai, China; 4 Institute of Respiratory Diseases, Shanghai Jiao Tong University School of Medicine, Shanghai, China; 5 Shanghai Key Laboratory of Emergency Prevention, Diagnosis and Treatment of Respiratory Infectious Diseases, Shanghai, China; 6 Department of Emergency, Ruijin Hospital, Shanghai Jiao Tong University School of Medicine, Shanghai, China

**Keywords:** Immunology, Cell biology

Dear Editor,

As of December, 2021, more than 272 million people have been infected with SARS-CoV-2. Multiple types of vaccines have been used to build herd immunity for the pandemic; however, decreased protective effect has been reported, and neutralizing antibody titers induced by the two doses of vaccination decline to near or below the seropositive threshold after 6 months^1^, indicating that the current COVID-19 vaccines provide relatively short-duration protection. In addition, with the unprecedented transmission of SARS-CoV-2, several more contagious Variants of Concern (VOCs) have emerged. Most recently, the B.1.1.529 variant Omicron, which was identified in November 2021, has spread internationally. The Omicron variant is the fifth VOC designated by the World Health Organization, primarily due to numerous mutations in the spike glycoprotein, especially in the receptor-binding domain and N-terminal domain. As the Omicron variant is the most divergent variant so far, it may lead to escape from immunity induced by the existing COVID-19 vaccines, and cause a large number of breakthrough infections^2^.

Waning immunity and viral diversification both create the potential need for further booster vaccination; therefore, we administered a homologous booster dose of the BBIBP-CorV vaccine, 8–9 months after completing the priming two-dose vaccination schedule, to eligible healthcare workers in Shanghai Ruijin Hospital to investigate whether the newly identified Omicron variant can escape serum antibody neutralization induced by the booster vaccination. Serum specimens were obtained 28 days after the second dose, before and 28 days after the booster dose. We determined the serum neutralizing activity using a pseudovirus-based neutralization assay, and SARS-CoV-2-specific antibody level, which is thought to be a good surrogate for neutralizing antibodies, was also assessed using a chemiluminescence immunoassay. The details of the methods are described in the Supplementary Methods.

A total of 292 participants were included in this study, of whom 72 were male and 220 were female, with a median age of 39.00 years (interquartile range (IQR) 32.00–46.00) years (Supplementary Table S1). The baseline immune responses at 8–9 months after the priming vaccination with two doses were weak. Specific antibodies against SARS-CoV-2 could still be detected in 229 (78.42%) of 292 participants, but the median antibody level dropped from 31.98 (10.36–73.66) on day 28 after the two-dose vaccination to 3.63 (1.16–9.93) (Fig. 1a). Moreover, only 53 (18.15%) of 292 participants had quantifiable neutralizing antibodies over the period of 8–9 months, and the geometric mean titer (GMT) declined rapidly to below the lower limit of detection (Fig. 1b).

**Fig. 1:**
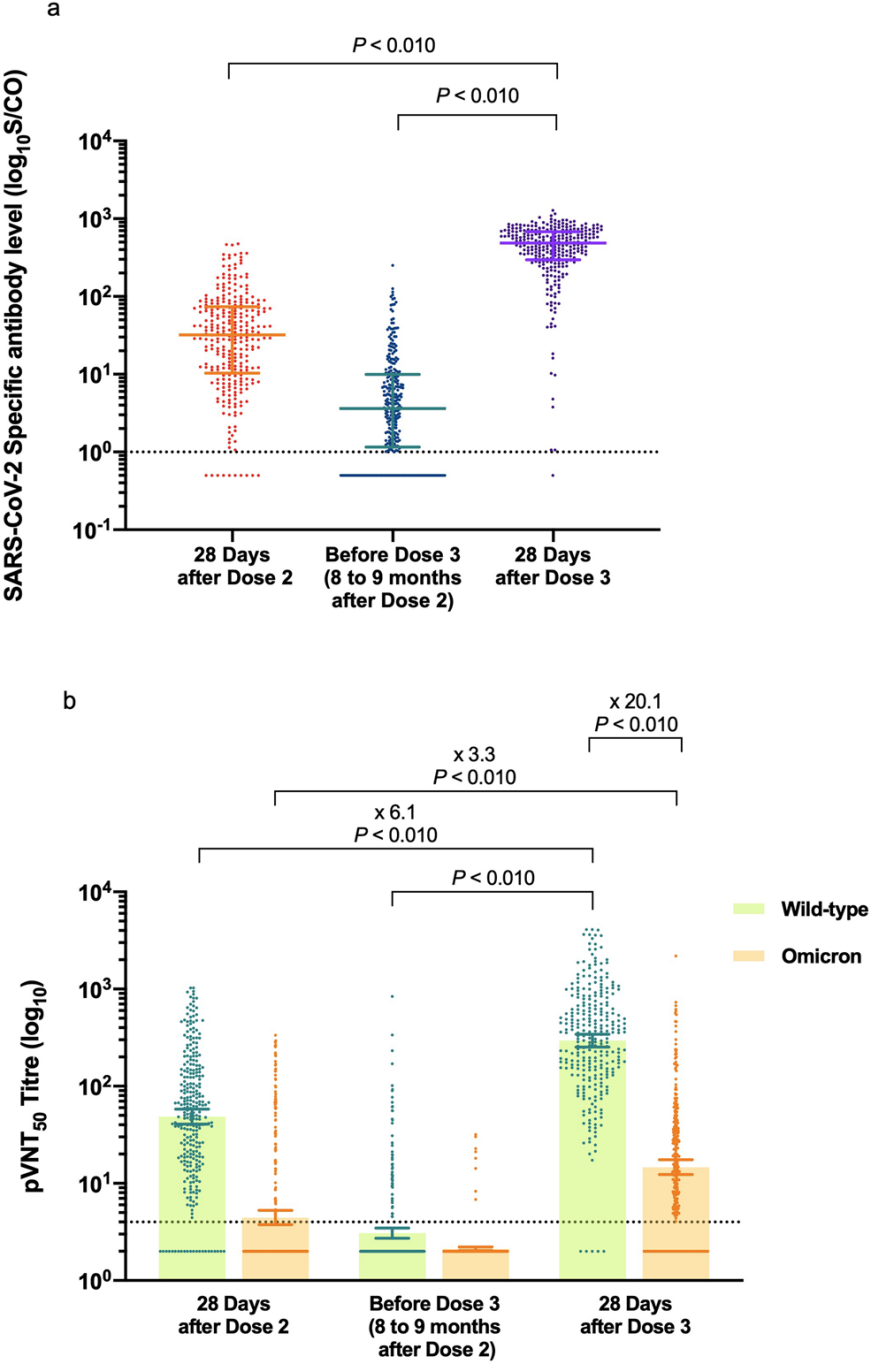
Anti-SARS-CoV-2-specific antibody and neutralizing antibody responses in serum samples of the 292 participants. **a** The specific antibody levels against SARS-CoV-2 at different time points are shown. The horizontal dashed line represents the lower limit of detection (LLD) of 1. Results below the LLD were set to 0.5 times the LLD. Data points shown on the bar graph represent individual titers. Error bars indicate median and interquartile range (IQR). **b** Results of 50% pseudovirus neutralization titer (pVNT50) against the WT strain and the Omicron variant at the time points are shown. The horizontal dashed line represents the lower limit of detection (LLD) of 4. Results below the LLD were set to 0.5 times the LLD. Data points shown on the bar graph represent individual titers. Error bars represent the geometric mean with the 95% confidence interval (95% CI). Fold-changes in geometric mean titer are shown above. *P* values were calculated using the Wilcoxon matched-pairs signed-rank test.

A significantly enhanced antibody response was observed on day 28 after the booster dose. Specific antibodies against SARS-CoV-2 were detected in 291 (99.66%) of 292 participants, with a median antibody level of 486.66 (296.16–681.91), which was markedly higher than the baseline antibody level and the level on day 28 after the second dose (Fig. 1a). The seroconversion rate of neutralizing antibodies against the wild-type (WT) strain was 98.29% (287/292), and the GMT increased to 294.85 (95% CI 252.99–343.65), >6.1 times of the level on day 28 after the second dose (Fig. 1b). The GMT on day 28 after the second dose was 48.65 (40.67–58.19), with 270 (92.47%) of 292 individuals showing detectable neutralizing activity.

On day 28 after the second dose, 75 (25.68%) of 292 vaccinated individuals displayed detectable serum neutralizing antibodies against the Omicron variant, resulting in a GMT of 4.45 (3.75–5.28). Over the period of 8–9 months, only eight out of 292 vaccinated individuals displayed quantifiable neutralizing antibodies against Omicron variant before the booster dose. Notably, on day 28 after the booster dose, 228 (78.08%) participants had neutralizing activity against the Omicron variant, and the booster dose resulted in an ~3.3-fold increase in neutralizing activity against the Omicron variant compared with the second vaccination, although the GMT showed a 20.1-fold reduction to 14.66 (12.30–17.48) relative to the WT strain (Fig. 1b), demonstrating that the Omicron variant exhibits escape capacity from neutralization induced by the booster dose. Additionally, sex and age were not factors that associated with the induction of neutralizing antibody and neutralizing titers against SARS-CoV-2 and the Omicron variant after the booster dose.

The booster dose of either an inactivated vaccine or a heterologous recombinant protein subunit vaccine can rapidly recover the neutralizing immune response to SARS-CoV-2^3^, and elicit neutralizing antibodies against VOCs, including variants Beta and Delta^4^. This study aimed to determine whether a homologous inactivated vaccine booster can effectively activate specific immune responses to SARS-CoV-2, especially enhancing the neutralizing activity against the newly-emerged Omicron variant. The data revealed that ~8–9 months after priming with two doses of inactivated vaccine, the neutralizing activity declined rapidly and could hardly be detected, supporting the need for a third dose to extend the duration of the humoral immune response against the emerging variants. As expected, a third dose following the priming with two doses of inactivated vaccine significantly recalled and enhanced antibody responses, indicating that the priming vaccination could induce efficient memory humoral immune responses. The neutralization GMT against the WT strain on day 28 after the third dose was 6.1 times higher than the GMT on day 28 after the second dose, but the persistence of the enhanced immunity against SARS-CoV-2 and its variants induced by a booster vaccination remains to be evaluated.

It has been suggested that achieving a higher neutralizing antibody titer with a booster dose is desirable to increase the breadth of neutralization^5^. However, based on the current data, sera from convalescent individuals^6^ and individuals who received two doses of vaccine^7^ had less neutralizing activity against the Omicron variant than against any other VOCs, including variants Beta and Delta. The substantial decrease in neutralizing activity in recipients of both homologous ChAd and BNT courses^8^, and a 41-fold decline in neutralization titers from recipients with or without previous SARS-CoV-2 infection^9^ suggest that the Omicron variant may escape from immune protection elicited by previous SARS-CoV-2 infection and by vaccination with existing COVID-19 vaccines. In our study, a homologous inactivated vaccine booster significantly improved the humoral immune response against the Omicron variant, which might be associated with the higher magnitude of WT neutralization, although the neutralizing activity was much less effective against the Omicron variant, with an ~20.1-fold reduction in neutralization titers relative to the WT strain. The results were consistent with a recent study, in which a significant reduction of neutralization titers against the Omicron variant was also observed post the homologous or heterologous booster vaccination in a relatively small sample size (*n* = 20)^10^. As neutralization is only a part of the immune response, and neutralizing activity does not reflect all potentially protective immune responses, real-world studies regarding the protection efficacy of the booster vaccination against the Omicron variant are required.

In conclusion, a booster dose of BBIBP-CorV led to a significant rebound in neutralizing immune response against SARS-CoV-2, while the Omicron variant showed extensive but incomplete escape from booster-enhanced neutralization. In current situation with the Omicron variant causing a rapidly increasing number of infections, the data presented here contribute evidence toward establishing a booster vaccination strategy against COVID-19.

## Supplementary information

Supplementary Information

## Data Availability

The data that support the findings of this study are available from the corresponding authors on reasonable request.

## References

[1] Zeng, G. et al. Immunogenicity and safety of a third dose of CoronaVac, and immune persistence of a two-dose schedule, in healthy adults: interim results from two single-centre, double-blind, randomised, placebo-controlled phase 2 clinical trials. *Lancet Infect. Dis.*10.1016/S1473-3099(21)00681-2 (2021). 10.1016/S1473-3099(21)00681-2 PMC8651254 34890537

[2] Pulliam, J. R. C. et al. Increased risk of SARS-CoV-2 reinfection associated with emergence of the Omicron variant in South Africa. *medRxiv*, 10.1101/2021.11.11.21266068 (2021).

[3] Cao, Y. et al. Humoral immunogenicity and reactogenicity of CoronaVac or ZF2001 booster after two doses of inactivatedvaccine. *Cell Res*. **32**, 107–109 (2021). 10.1038/s41422-021-00596-5 PMC8640508 34862467

[4] Ai, J. et al. Recombinant protein subunit vaccine booster following two-dose inactivated vaccines dramatically enhanced anti-RBD responses and neutralizing titers against SARS-CoV-2 and Variants of Concern. *Cell Res*. **32**, 103–106 (2021). 10.1038/s41422-021-00590-x PMC8609258 34815511

[5] Falsey AR (2021). SARS-CoV-2 neutralization with BNT162b2 vaccine dose 3. N. Engl. J. Med..

[6] Zhang, L. et al. The significant immune escape of pseudotyped SARS-CoV-2 Variant Omicron. *Emerg. Microbes. Infect.* **11**, 1–5 (2021). 10.1080/22221751.2021.2017757 PMC8725892 34890524

[7] Lu, L. et al. Neutralization of SARS-CoV-2 Omicron variant by sera from BNT162b2 or Coronavac vaccine recipients. *Clin. Infect. Dis.*10.1093/cid/ciab1041 (2021). 10.1093/cid/ciab1041 PMC8754807 34915551

[8] Dejnirattisai, W. et al. Reduced neutralisation of SARS-CoV-2 omicron B.1.1.529 variant by post-immunisation serum. *Lancet*, **399**, 234–236 (2021). 10.1016/S0140-6736(21)02844-0 PMC8687667 34942101

[9] Cele, S. et al. SARS-CoV-2 Omicron has extensive but incomplete escape of Pfizer BNT162b2 elicited neutralization and requires ACE2 for infection. *medRxiv*, 10.1101/2021.12.08.21267417 (2021).

[10] Ai, J. et al. Omicron variant showed lower neutralizing sensitivity than other SARS-CoV-2 variants to immune sera elicited by vaccines after boost. *Emerg. Microbes. Infect.* 1–24, 10.1080/22221751.2021.2022440 (2021). 10.1080/22221751.2021.2022440 PMC8788341 34935594

