# Supplementary material for: Reduced sensitivity of SARS-CoV-2 Omicron variant to antibody neutralization elicited by booster vaccination
Source: Cell Discov. 2022 Jan 17;8:4. doi: 10.1038/s41421-022-00375-5 (PMC8761745; doi:10.1038/s41421-022-00375-5)
Supplement: Supplementary file 1 — Supplementary Information [file 41421_2022_375_MOESM1_ESM.pdf]

Supplementary Table S1. Basic Characteristics of participants

	Vaccine recipients (n = 292)
Age	39.00 (32.00-46.00)
Sex	
Male	72 (24.66%)
Female	220 (75.34%)
Underlying diseases	19 (6.51%)
Diabetes	2 (0.68%)
Hypertension	9 (3.08%)

Data are expressed as median (interquartile range [IQR]) or number (%).

Supplementary Methods.

Participants and blood samples

Eligible participants for the third booster dose were healthcare workers in Shanghai Ruijin Hospital aged 18-59 years, who had received priming vaccination with two doses, 21 days apart of inactivated SARS-CoV-2 vaccine (BBIBP-CorV, Sinopharm) at least 8 months previously. Blood samples were taken from 292 participants for serology tests before and 28 days after the third booster dose. The protocol and informed consent were approved by the Ethics Committee of Shanghai Ruijin Hospital (RJHKY2021-321) in accordance with the Declaration of Helsinki and Good Clinical Practice. Written informed consent was obtained from all participants.

SARS-CoV-2 specific antibody response assessment

The specific antibodies against SARS-CoV-2 were measured using a chemiluminescence kit manufactured by Wantai BioPharm (China). The antibody levels were expressed by the chemiluminescence signal according to the manufacturer's instructions.

Pseudovirus based neutralization assay

A pseudovirus-based neutralization assay was performed as previously described[1] with minor modifications. In brief, the lentivirus-based SARS-CoV-2 pseudoviruses expressing a luciferase reporter gene and bearing the spike protein from the wild-type strain (Wuhan-Hu-1) or the Omicron variant (lineage B.1.1.529) tested in this study were manufactured by Vazyme Biotech Co., Ltd (China). The 50% tissue culture infectious dose ($TCID_{50}$) of the lentivirus-based SARS-CoV-2 pseudoviruses was measured by a luciferase assay in relative light units (RLUs) and calculated according to the Reed-Muench method, and the SARS-CoV-2 pseudoviruses were diluted to the same amount (10,000 $TCID_{50}$ per mL) for use. Vaccine-elicited sera were inactivated at 56°C for 30 mins before assessing the neutralization geometric mean titers (GMTs). Six

serial dilutions of heat-inactivated sera (in a four-fold step-wise manner, initially 1:4 diluted) were incubated with 250 $TCID_{50}$ SARS-CoV-2 pseudoviruses per well for 1 hour, together with the virus control and cell control wells, before seeding 20,000 HEK293T-ACE2 cells per well in 96-well plates. Following 48 hours of incubation in a 5% CO_2 environment at 37℃, the supernatant was removed, and the luminescence were measured using Luciferase Assay System (Promega Biotech Co., Ltd) according to the manufacturer's instructions. The 50% inhibitory concentration (IC_{50}) was defined as the serum dilution at which the relative light units (RLUs) were reduced by 50% compared with the virus control wells after subtraction of the background RLUs in the cell control wells with cells only. The IC_{50} values were calculated by generating a three-parameter non-linear regression curve fit in GraphPad Prism 8.4.0. Neutralizing antibody potency < 1:4 was considered negative. The neutralizing titer for each sample was measured twice in two independent experiments.

Statistical analysis

Continuous variables that were not normally distributed were presented as median (interquartile range [IQR]). Categorical variables were described as count (%). Antibody titers were reported as the geometric mean titer (GMT) with the corresponding 95% confidence intervals (95% CI). The values were compared using the Wilcoxon matched-pairs signed-rank test. Graphs were plotted using GraphPad Prism 8.4.0. Statistical analyses were performed using SPSS 24.0. Two-sided *P* values of less than 0.050 were considered statistically significant.

References

1. Nie, J., *et al.* Quantification of SARS-CoV-2 neutralizing antibody by a pseudotyped virus-based assay. *Nat Protoc* **15**, 3699-3715 (2020).
